# IL-2/Anti-IL-2 Complex Attenuates Inflammation and BBB Disruption in Mice Subjected to Traumatic Brain Injury

**DOI:** 10.3389/fneur.2017.00281

**Published:** 2017-06-30

**Authors:** Weiwei Gao, Fei Li, Ziwei Zhou, Xin Xu, Yingang Wu, Shuai Zhou, Dongpei Yin, Dongdong Sun, Jianhua Xiong, Rongcai Jiang, Jianning Zhang

**Affiliations:** ^1^Department of Neurosurgery, Tianjin Medical University General Hospital, Tianjin Neurological Institute, Key Laboratory of Post-Neurotrauma, Neuro-Repair and Regeneration in the Central Nervous System, Ministry of Education and Tianjin City, Tianjin, China

**Keywords:** traumatic brain injury, IL-2/anti-IL-2, regulatory T cells, microglia, inflammation, blood–brain barrier

## Abstract

Traumatic brain injury (TBI) induces the excessive inflammation and disruption of blood–brain barrier, both of which are partially mediated by the activation of microglia and release of inflammatory cytokines. Previous reports showed that administration of regulatory T cells (Tregs) could suppress inflammation and promote neurological function recovery, and that the IL-2/anti-IL-2 complex (IL-2C) could increase the number of Tregs. Thus, we hypothesized that IL-2C-mediated expansion of Tregs would be beneficial in mice subjected to TBI. In this study, mice received an intraperitoneal injection of IL-2C for three consecutive days. We observed that IL-2C dose-dependently increased Tregs without affecting the populations of CD4, CD8, or natural killer cells. IL-2C could improve the neurological recovery and reduce brain edema, tissue loss, neutrophils infiltration, and tight junction proteins degradation. Furthermore, this complex could also reduce the expression of CD16/32, IL-1β, or TNF-α, and elevate the expression of CD206, arginase 1, or TGF-β. These results suggest that IL-2C could be a potential therapeutic method to alleviate excessive inflammation and maintain blood vessel stability after TBI.

## Introduction

Traumatic brain injury (TBI) is one of the leading causes of death and severe disability worldwide, especially in the young people. Although many interventions are applied at different time points post-injury, it remains a major challenge to promote long-term recovery in these patients. Following the initial trauma, inflammatory responses can aggravate brain damage, mainly *via* the infiltration of leukocytes and the activation of microglia (1–4). Although these cells are essential for debris clearance and tissue remodeling (5), their overactivation results in the release of a large number of cytotoxic molecules that eventually damage neurons, the extracellular matrix, and endothelial cells (ECs) (6, 7).

Tregs are key endogenous immune-regulatory cells that regulate the inflammatory response (8–10). An *in vitro* experiment showed that Tregs inhibited the pro-inflammatory effects of macrophages and promoted macrophage differentiation toward an anti-inflammatory phenotype (11). Liesz et al. showed that boosting Tregs after stroke reduced microglial cell activation and neurotoxic cytokine secretion (12, 13). Furthermore, Li et al. demonstrated that the intravenous transfer of Tregs exerts a neuroprotective effect by suppressing neutrophil-derived matrix metallopeptidase 9 and reducing the subsequent proteolytic damage of the blood–brain barrier (14). However, few studies examined the effects of Tregs in TBI except a report by Yu et al. that deleting Tregs accelerated the damage caused by TBI and adoptive transfer exerted a suppressive effect on inflammatory reactions (15). It, therefore, seems that increasing the level of Tregs following TBI may be a potential strategy to improve prognoses in these patients. IL-2/anti-IL-2 complex (IL-2C) has been reported to rapidly increase Tregs in different diseases, including stroke, myasthenia, or atherosclerosis, and provides protective effects by inhibiting inflammation and substantially attenuating the progression of these diseases (12, 16–19). The potential mechanism is that anti-IL-2 (JES6-1) prevent interaction of IL-2 with IL-2 receptor β-chain without affecting IL-2 receptor α-chain (CD25) (18). However, the effects of IL-2C on TBI remain unexplored. Thus, in present study, we aimed to explore the role of IL-2C treatment on recovery after TBI.

## Materials and Methods

### Animals and TBI Model

Male C57BL/6 mice (20–23 g) were obtained from the Experimental Animal Laboratories of the Academy of Military Medical Sciences and housed individually in a temperature- (22°C) and humidity-controlled (60%) vivarium with free access to food and water. All experimental procedures were approved by the Chinese Small Animals Protection Association Experimental Protocol.

In this study, a total of 105 male mice were included in this study and divided into the following three groups (*n* = 35/group): group I, mice subjected to TBI and intraperitoneally injected with IL-2C; group II, mice subjected to TBI and injected with an equal volume of PBS; and group III, mice subjected to sham surgery only. For each, five mice were evaluated for beam walk/forelimb foot faults, flow cytometry, tissue loss, immunohistochemistry, water content, immunobloting for selective markers, and immunofluorescence.

The TBI model was constructed using a controlled cortical impact (CCI) device (eCCI Model 6.3; VCU, Richmond, VA, USA). Briefly, anesthetized mice were placed in a stereotaxic frame, and a 2.0-mm hole was drilled in the right parietal skull to expose the dura. Then, the mice received a single impact with an impact velocity of 4.5 m/s and a dwell time of 150 ms to cause a 1.5-mm-deep deformation in the parietal association cortex. Sham injuries were performed without the impact.

### IL-2C Administration

**Figure 1:**
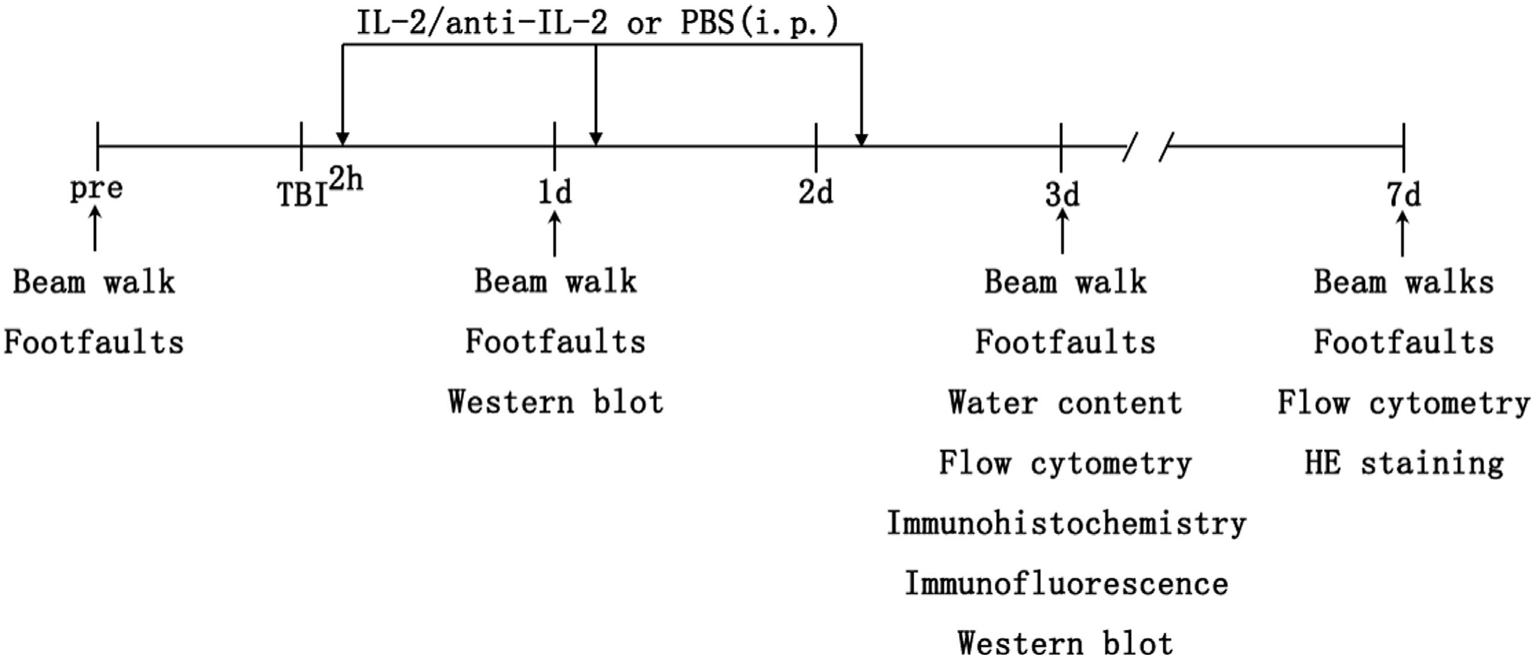
Schematic diagram of the experimental design. C57BL/6 mice underwent controlled cortical impact to the right cortex on day 0. Then, the mice received 200 µl intraperitoneal injections of either IL-2C or PBS at 2, 24, and 48 h after traumatic brain injury (TBI). The brains were harvested post-injury for immunohistochemistry/immunofluorescence, flow cytometry, brain water content, and protein analysis. Sham mice were subjected to the same assays without undergoing brain injury.

IL-2/anti-IL-2 complexes were prepared as previously described (20). Recombinant mouse IL-2 (rmIL-2, Cat #: 575406) and purified anti-IL-2 (Cat #: 503704) were purchased from BioLegend (SanDiego, CA92121). Mice subjected to TBI were intraperitoneally injected with 200 µl of either IL-2C or PBS at 2, 24, and 48 h after TBI (Figure 1). IL-2C was mixed with rmIL-2 and anti–IL-2 as follows: (1) IL-2C^1^, 1 µg IL-2 + 5 μg anti-IL-2; (2) IL-2C^2^, 0.5 µg IL-2 + 2.5 μg anti-IL-2; and (3) IL-2C^3^, 0.1 µg IL-2 + 0.5 μg anti-IL-2. Before the complexes were injected, they were incubated at 37°C for 30 min.

### Isolation of Mononuclear Cells from the Brain

Mononuclear cells were isolated from the nervous system using discontinuous Percoll gradients (21). After five mice were transcardially perfused with cold PBS, the cerebral cortices ipsilateral to injury (hereafter referred to as ipsilateral cortices) were collected and mechanically homogenized through a 40-µm cell strainer. The cells were washed with cold PBS in a 50-ml tube and centrifuged at 2,000 rpm for 5 min at 4°C. The resulting cell pellets were resuspended in 5 ml of 30% Percoll (Sigma Aldrich, Cat #: P1644) and centrifuged against 70% Percoll in a 15-ml tube for 15 min. The cell monolayer between the 30 and 70% Percoll interfaces was collected and washed once for further staining.

### Flow Cytometry

Mononuclear cells from the spleens and brains were surface-labeled with anti-mouse CD4 FITC (eBioscience, Cat #: 11-0042), CD25 PE (eBioscience, Cat #: 12-0251), NK1.1 PE (eBioscience, Cat #: 12-5941-82), CD8 FITC (eBioscience, Cat #: 11-0081-81), and B220 APC (eBioscience, Cat #: 17-0452-81). For Tregs labeling, the cells were further fixed and permeabilized using a Foxp3/transcription Factor Staining Buffer kit (eBioscience, Cat #: 00-5523) and then stained with anti-mouse Foxp3 APC (eBioscience, Cat #: 17-5773). Cells were washed and suspended in flow cytometry staining buffer (eBioscience, Cat #: 00-4222-57). FACS analysis was performed using Accuri C6 software (BD Biosciences, San Jose, CA, USA).

### Tissue Loss

Plenty of blood and necrotic tissue around the traumatic lesion on day 3 after TBI, which would affect the experimental results. Thus, we randomly selected five mice sacrificed and transcardially perfused with saline and 4% paraformaldehyde on day 7 after TBI. After dehydration and transparency, the brains were embedded in the paraffin and cut into 6 µm sections. To assess the lesion volume, we prepared seven brain sections, taken every 0.5 mm from 0.5 to 3.5 mm posterior to bregma, which were stained with Gill’s hematoxylin and eosin. The images were captured using an inverted fluorescence microscope (Olympus, Japan) and digitalized with ImageJ (NIH, Bethesda, MD, USA). We calculated the lesion area of each section by subtracting the size of hemisphere ipsilateral to injury (hereafter referred to as ipsilateral hemisphere) from the hemisphere contralateral to injury (hereafter referred to as contralateral hemisphere). Then, the lesion volume was computed by integrating the lesion area of each section and the distance between two sections. Tissue loss in the ipsilateral hemisphere was calculated as a percentage of the contralateral hemispheric volume.

### Immunohistochemistry

After the brain sections were deparaffinized and rehydrated, they were boiled in citrate buffer (pH 6.0) for antigen recovery, incubated with 3% hydrogen peroxide (H_2_O_2_) for 20 min, and then 3% bovine serum albumin in PBS for 60 min (to block non-specific binding). After pretreatment, the sections were incubated overnight with rabbit anti-mouse MPO antibodies (dilution 1:100, Abcam, Cat #: ab9535), rabbit anti-mouse Iba-1 antibodies (dilution 1:500, Wako, Cat #: 019-19741), and goat anti-arginase 1 (dilution 1:100, Santa Cruz Biotechnology, Cat #: sc-18355). They were then incubated with biotinylated anti-rabbit immunoglobulin G (dilution 1:400, Vector) for 2 h at RT. The bound antibody was recognized using an avidin-peroxidase conjugate solution (ABC, 1:1000; Vector) and visualized using diaminobenzidine. The negative control slices were treated with the same immunoblotting procedures but without the primary antibody.

### Immunofluorescence

Immunofluorescence was performed as previously described (22). On day 3 post-injury, five mice were euthanatized to prepare frozen brain sections. The embedded brains were cut into 6 µm-thick sections, fixed in acetone at 4°C for 20 min, and incubated in 3% BSA for 30 min at 37°C. The sections were then incubated with the following antibodies overnight: anti-CLN5 (1:100, Abcam, Cat #: ab170889), anti-Iba-1 (1:500, Wako, Cat #: 019-19741) in combination with anti-CD16/32 (1:250, BD Pharmingen, Cat #: 553140) or anti-CD206 (1:100, R&D system, Cat #: AF2535) antibodies. The sections were then incubated with Alexa Fluor-conjugated anti-rabbit or anti-mouse IgG (1:500; molecular probes) antibodies at RT for 1 h in the dark. Cell nuclei were counterstained with DAPI. The sections were observed using a fluorescence microscope.

### Western Blot Analysis

Western blot analysis was performed as previously described (22). Total protein from five mice was extracted from the ipsilateral cortices using ice-cold RIPA buffer. Protein concentrations were detected using BCA Protein Assay Kits (Thermo Fisher). Protein samples were separated using sodium dodecyl sulfate-polyacrylamide gel electrophoresis and then transferred to polyvinylidene difluoride membranes. The relevant proteins were detected by incubating the membranes with primary antibodies overnight (i.e., β-actin, 1:1,000, CST, Cat #: 4970S; TNF-α, 1:10,000, GeneTex, Cat #: GTX110520; IL-1β, 1:1,000, CST, Cat #: 12242S; TLR4, 1:1,000, Abcam, Cat #: ab13556; NF-κB, 1:1,000, CST, Cat #: 8482S; TGF-β, 1:2,000, Torrey Pines Biolabs; ZO-1, 1:1,000, Invitrogen, Cat #: 40-2200; and occludin, 1:1,000, Invitrogen, Cat #: 33-1500) and then with secondary antibodies (i.e., horseradish-peroxidase conjugated goat anti-rabbit or anti-mouse IgG, 1:3,000, Cell Signaling Technology). Immunoblots were visualized using a Millipore ECL Western Blotting Detection System (Millipore, Billerica, MA, USA).

### Brain Water Content

Brain water content was used to assess brain edema. Five mice were sacrificed on day 3, the time point associated with maximal edema following TBI (23). Following sacrifice, the brains were divided along the midline, and the ipsilateral hemispheres were immediately weighed to obtain a wet weight (WW). The tissues were then dried at 60°C for 72 h and weighed to obtain a dry weight (DW) (24). The water content of each sample (% water content) was calculated using the following formula: (WW−DW)/WW × 100%.

### Beam Walking and Forelimb Foot Faults

In the motor function test, the mice were examined for fine motor function using a beam walking task with an elevated narrow beam (80 cm long × 2.5 cm wide) raised to 10 cm above ground level. The time to traverse the beam was recorded and analyzed after three trials (60 s allotted time). Animals were pre-trained for 3 days before CCI.

The forelimb foot faults were used to determine forelimb movement dysfunction. Mice from different groups were placed on the elevated horizontal grids. Mice placed their paws on the wire while moving along the grid. With each step, the paw may fall or slip between wires. If this occurred, it was recorded as a foot fault. A total of 50 steps were recorded for the right forelimb. And the total number of foot faults was recorded as described. Mice were trained for 3 days before CCI, and the number of foot faults following CCI at days 0, 3, 7, and 14 was recorded.

### Statistical Analysis

All data are presented as the mean ± SD. For comparisons among multiple groups, one-way analysis of variance (ANOVA) followed by a *post hoc* (Bonferroni) test was used to determine significant differences. Differences between two groups were analyzed using Student’s *t*-test. A repeated-measures ANOVA (RM ANOVA) was used for beam walks and forelimb foot faults. A value of *p* < 0.05 was considered statistically significant. All analyses were performed using SPSS statistical software (version 19.0; IBM Corporation, Armonk, NY, USA).

## Results

### Effects of Different Doses of IL-2C on TLR4/NF-κB Expression and the Number of Treg Cells

**Figure 2:**
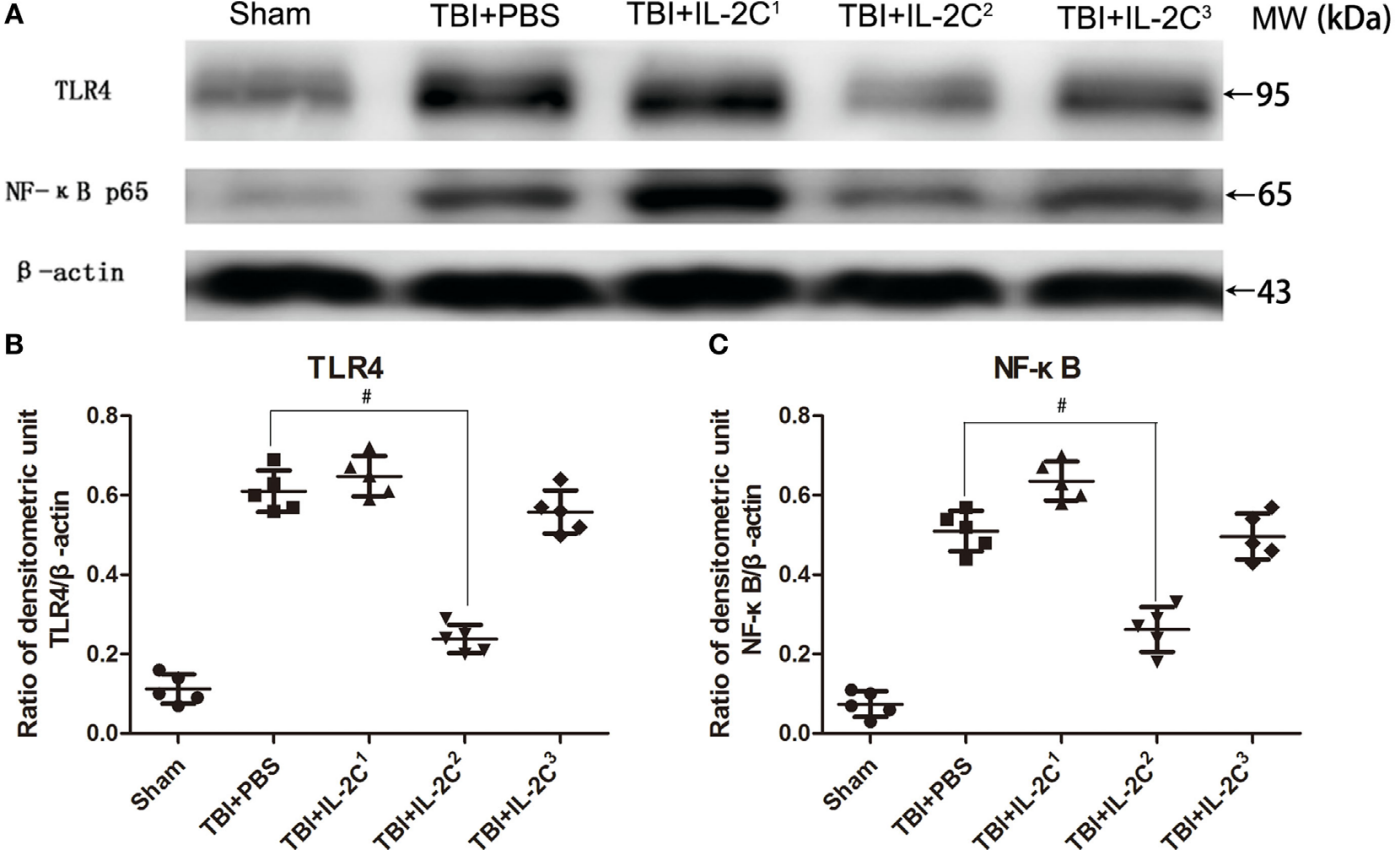
**(A)** Representative immunoblot showing TLR4 and NF-κB expression on day 3 post-injury in the ipsilateral cortices of mice treated with either PBS or different doses of IL-2C, as indicated. **(B,C)** Quantitative evaluation showed that IL-2C^2^ (0.5 µg IL-2 + 2.5 μg anti-IL-2) significantly reduced the expression of both inflammatory factors. Data are presented as the mean ± SD; *p < 0.05 and ^#^*p* < 0.01.

In this study, we administered the TBI mice three different doses to discern the optimal dosage. The results indicated that IL-2C^2^ (0.5 µg IL-2 ± 2.5 μg anti-IL-2) most efficiently reduced TLR4 and NF-κB expression in the ipsilateral cortices. The higher and lower dose had no effect on the expression of inflammation-associated proteins (Figure 2). We, therefore, chose this dose as the optimal concentration for subsequent experiments.

**Figure 3:**
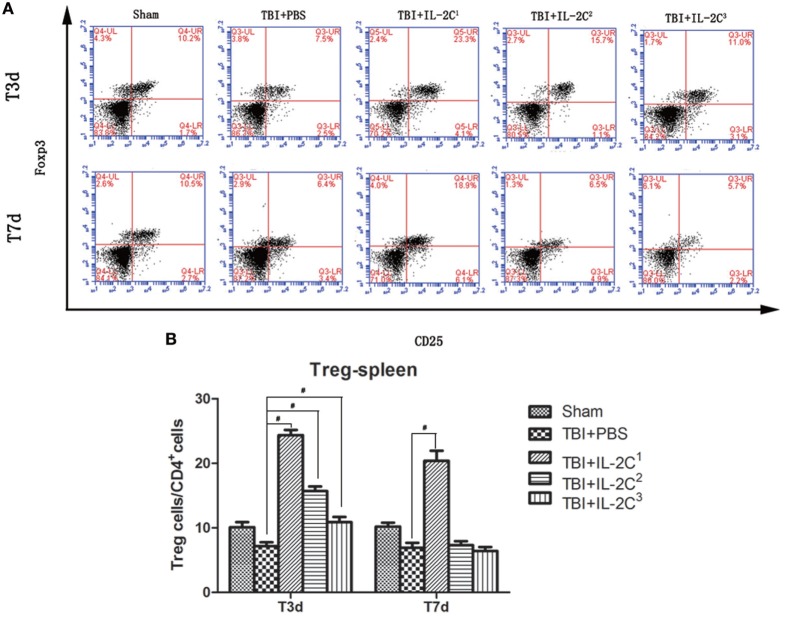
**(A)** IL-2C administration dose-dependently increased the number of CD4^+^ CD25^+^ Foxp3^+^ T cells in the spleen. Plots represent the percentage of CD25^+^ Foxp3^+^ cells within the CD4^+^ population. **(B)** Cell count analysis indicated that three consecutive injections of the IL-2C increased the number of Tregs after 3 and 7 days. Data are presented as the mean ± SD; *p < 0.05 and ^#^*p* < 0.01 (*n* = 5 mice/group).

Next, we determined the number of Tregs in the spleen on day 3 and 7 after TBI using flow cytometry and found treatment with IL-2C dose-dependently increased the percentage of Tregs. The proportion of Tregs among the CD4^+^ cell population was as follows: for day 3, IL-2C^1^: 7.18 ± 0.59 versus 24.38 ± 0.82%, *p* < 0.01; IL-2C^2^: 7.18 ± 0.59 versus 15.72 ± 0.70%, *p* < 0.01; and IL-2C^3^: 7.18 ± 0.59 versus 10.88 ± 0.82%, *p* < 0.01. For day 7, 6.92 ± 0.77 versus 20.42 ± 1.54, *p* < 0.01; 6.92 ± 0.77 versus 7.34 ± 0.61, *p* > 0.05; and 6.92 ± 0.77 versus 6.44 ± 0.59, *p* > 0.05, respectively (Figure 3).

### Effects of IL-2C Administration on Peripheral CD4^+^, CD8^+^, and NK^+^ Cells

**Figure 4:**
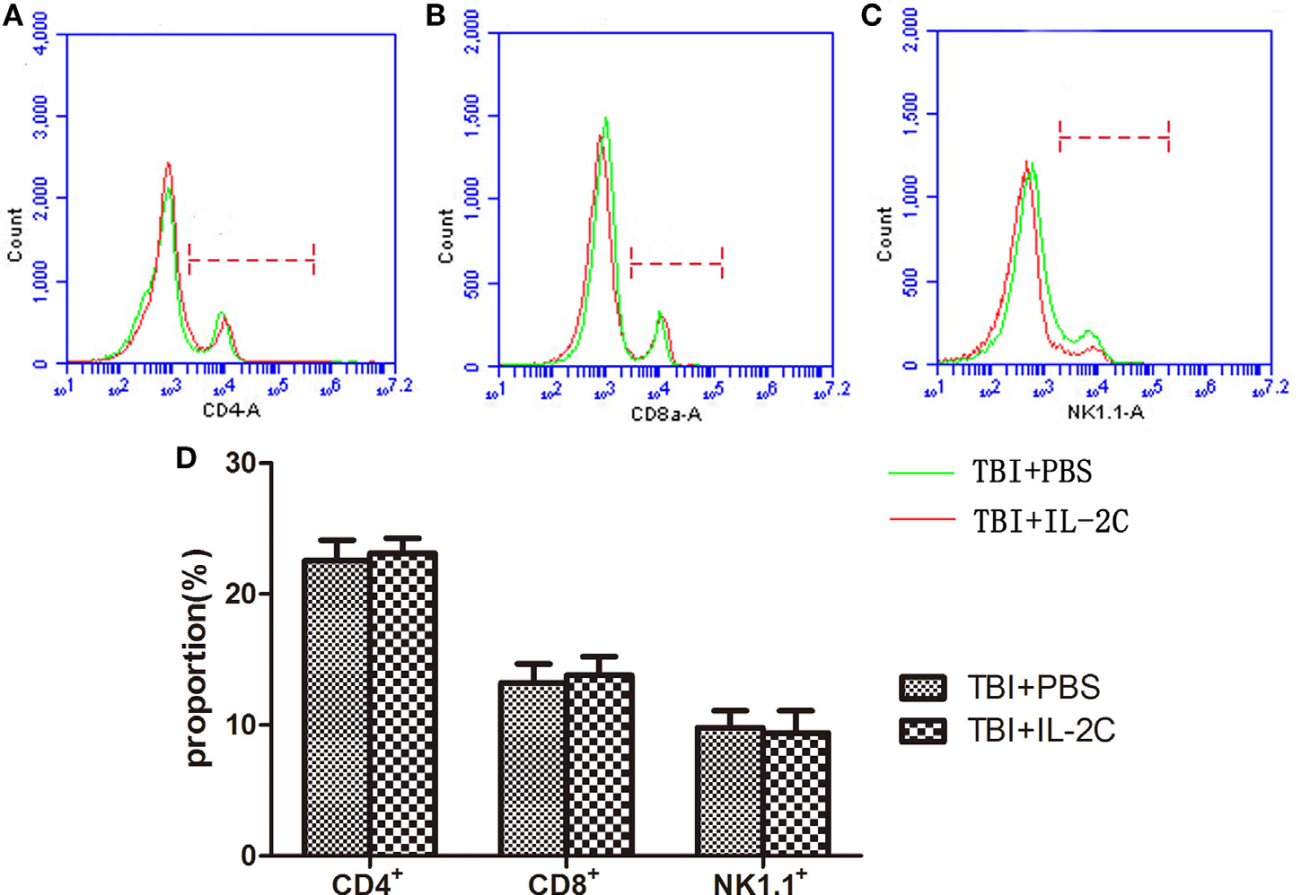
Flow cytometry was used to count the number of **(A)** CD4^+^, **(B)** CD8^+^, and **(C)** NK1.1^+^ cells isolated from the spleen on day 3 post-injury. We observed that treatment with IL-2C had no effect on the proportion of each lymphocyte subtype. **(D)** Bar graphs indicate that there was no difference between the PBS- and IL-2C-treated groups. Data are presented as the mean ± SD; *p < 0.05 and ^#^*p* < 0.01 (*n* = 5 mice/group).

We next sought to determine whether the IL-2C influences the population distribution of other lymphocytes. We isolated the mononuclear cell suspensions for flow cytometry analysis and found that the IL-2C selectively increased the number of Tregs without affecting CD4, CD8, or NK cells (13.215 ± 1.47 versus 13.785 ± 1.43, *p* > 0.05; 9.8 ± 1.29 versus 9.41 ± 1.68%, *p* > 0.05; and 50.52 ± 3.26 versus 52.88 ± 8.66%, *p* > 0.05, respectively) (Figure 4).

### Effects of IL-2C Administration on the Number of Tregs in the Brain

**Figure 5:**
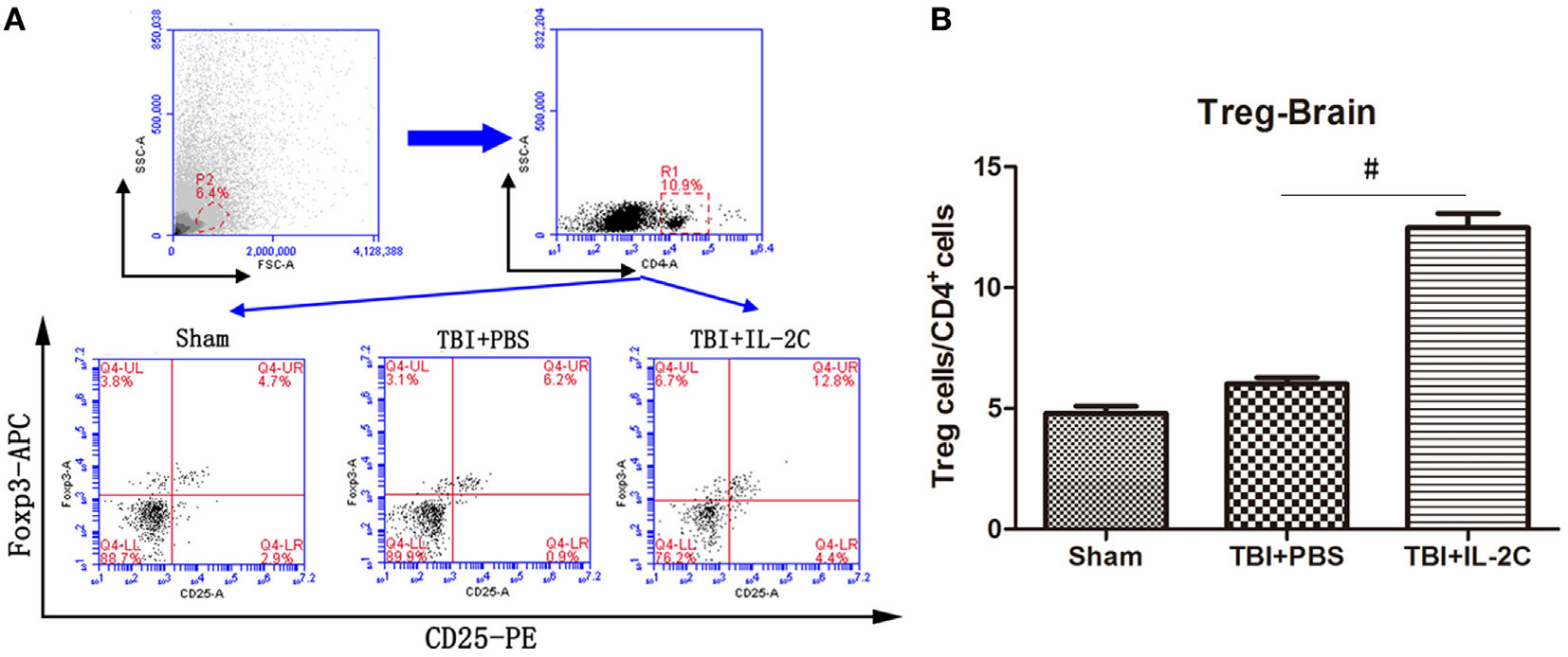
**(A)** On day 3 after traumatic brain injury (TBI), mononuclear cells were isolated from the brain using discontinuous Percoll gradients, and flow cytometry analysis was used to detect the percentage of Tregs within the CD4^+^ population. **(B)** Treatment with IL-2C significantly increased the number of Tregs in the central nervous system on day 3 post-injury. Data are presented as the mean ± SD; *p < 0.05 and ^#^*p* < 0.01 (*n* = 5 mice/group).

We isolated mononuclear cells from the ipsilateral cortices and assessed the number of Tregs using flow cytometry. We observed that IL-2C significantly increased the number of CD4^+^CD25^+^Foxp3^+^cells in the injured brain (6.02 ± 0.56 versus 12.48 ± 0.59, *p* < 0.01) (Figure 5).

### Effects of IL-2C Administration on the Brain Water Content after TBI

**Figure 6:**
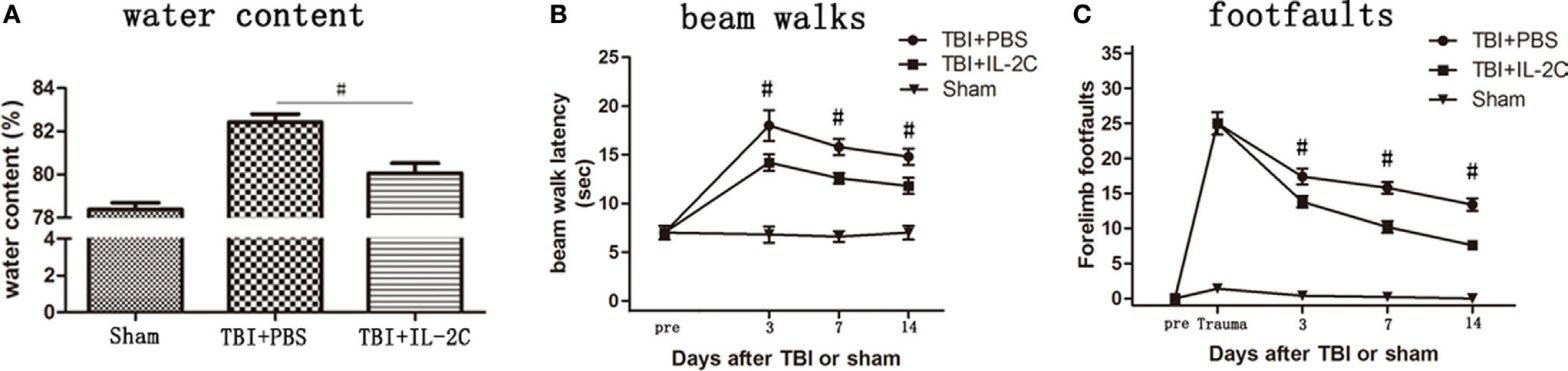
**(A)** The brain water content of the ipsilateral hemisphere was measured at 72 h after injury. Brain water content was significantly higher in the control than in the sham group, whereas treatment with IL-2C significantly reduced brain water content (*n* = 5 mice/group). **(B)** Treatment with IL-2C significantly improved the beam walking speed compared with the PBS group, measured until 14 days after traumatic brain injury (TBI). **(C)** IL-2C treatment significantly reduced the number of forelimb foot faults from day 3 after TBI (*n* = 5 mice/group). Data are presented as the mean ± SD; *p < 0.05 and ^#^*p* < 0.01.

To determine whether IL-2C administration reduced brain edema, we used dry-WW to measure brain water content in the experimental groups. Brain water content was significantly higher in the PBS-treated group (*p* < 0.01), and IL-2C administration significantly reduced brain water content (*p* < 0.01). The brain water content in the sham, PBS, and IL-2C groups was 78.38 ± 0.31, 82.42 ± 0.38, and 80.06 ± 0.46%, respectively (Figure 6A).

### Effects of IL-2C Administration on the Recovery of Neurological Function

To assess the effects of systemic administration of IL-2C on TBI mice, beam walks and forelimb foot faults were performed on days 3, 7, and 14 after TBI. Treatment with IL-2C significantly improved the beam walking speed compared with the PBS group, measured until 14 days after TBI (Figure 6B, *p* < 0.01). Then, IL-2C treatment significantly reduced the amount of forelimb foot faults at all the recorded time points post-injury (Figure 6C, *p* < 0.01).

### Effects of IL-2C Administration on the Cortical Contusion Volume

**Figure 7:**
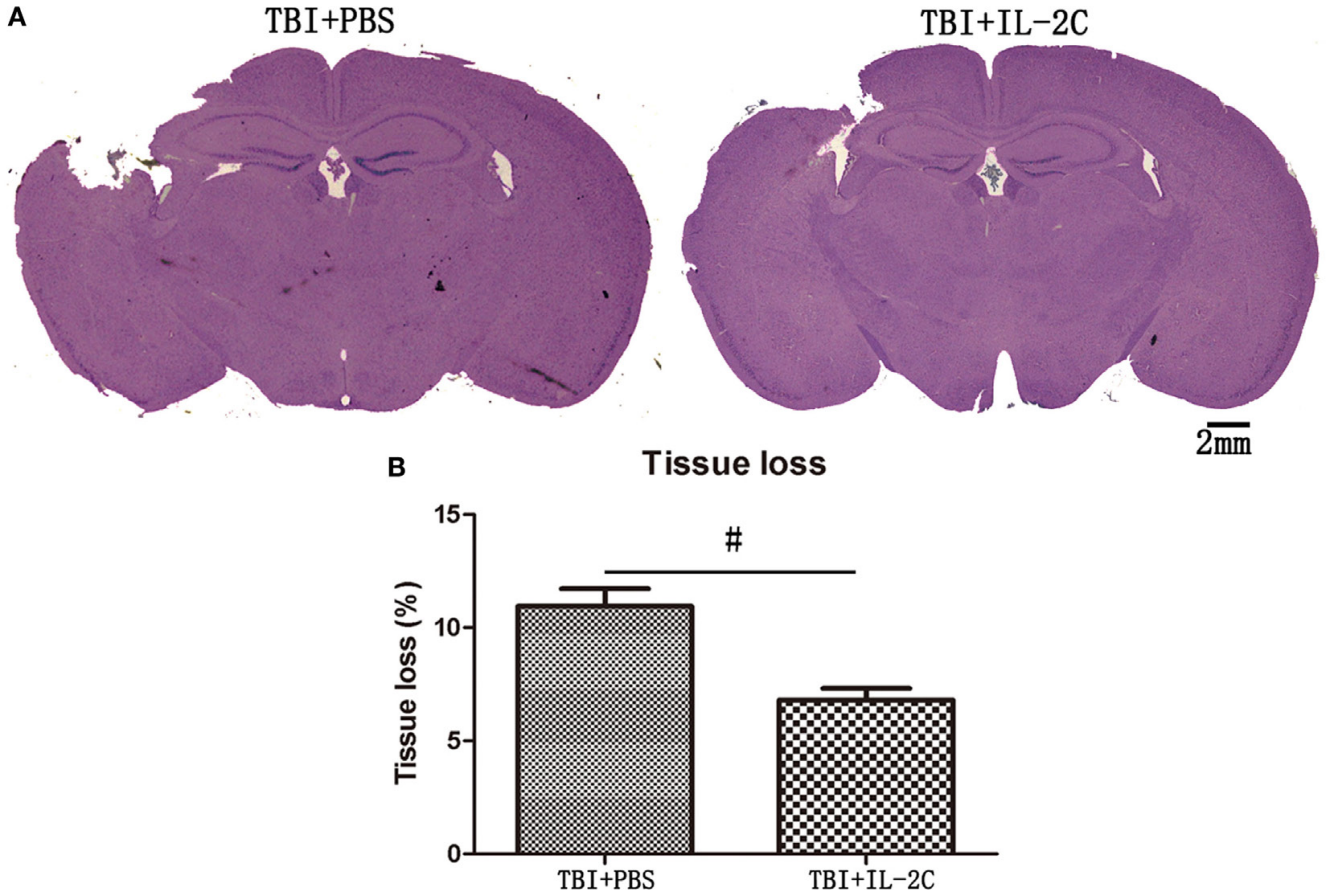
**(A)** Representative Mayer’s Hematoxylin and Eosin-stained brain sections obtained from PBS and IL-2C-treated mice at 7 days post-traumatic brain injury (TBI) (scale bar = 2 mm). **(B)** The bar graph shows that treatment with IL-2C resulted in significantly less tissue loss than was observed in the control (*n* = 5 mice/group). Data are presented as the mean ± SD; *p < 0.05 and ^#^*p* < 0.01.

To determine the impact of IL-2C on injury size later during disease progress, we compared tissue loss in the injured hemisphere to that observed in the uninjured hemisphere. We observed that there was a significantly less ipsilateral hemisphere tissue loss in the treated mice than in the injured control mice on day 7 after injury (10.94 ± 0.78 versus 6.80 ± 0.51%, *p* < 0.01) (Figure 7).

### Effects of IL-2C Administration on the Integrity of the Blood–Brain Barrier

**Figure 8:**
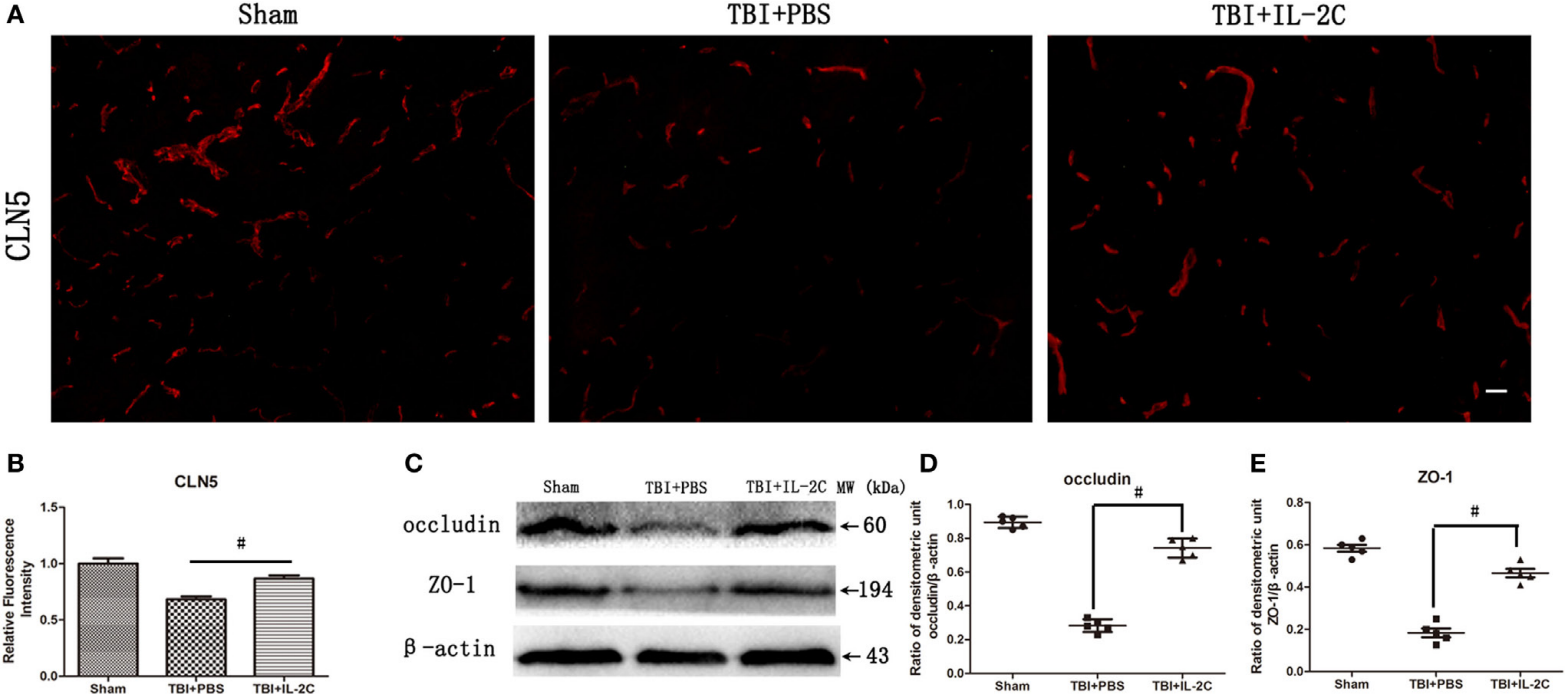
**(A,B)** Immunofluorescent staining and relative fluorescence intensity showed that treatment with IL-2C retained the continuity of the tight junction protein (TJ) CLN5. **(C–E)** IL-2C administration significantly increased the expression of TJs occludin and ZO-1 compared to the PBS treated group. Data are presented as the mean ± SD; *p < 0.05 and ^#^*p* < 0.01 (*n* = 5 mice/group).

Tight junction proteins (TJs) are expressed in a continuous manner in intact animals but are largely disrupted in injured areas 24 h after TBI. In this study, immunofluorescence showed that IL-2C therapy retained the integrity of CLN5 expression (Figures 8A,B). Moreover, we measured the expression of TJs using western blot, and IL-2C treatment increased the levels of the TJs ZO-1 (0.47 ± 0.05 versus 0.15 ± 0.04) and occludin (0.66 ± 0.05 versus 0.31 ± 0.03), respectively (Figures 8C–E).

### Effects of IL-2C Administration on the Infiltration of Neutrophils and Activation of Microglial

**Figure 9:**
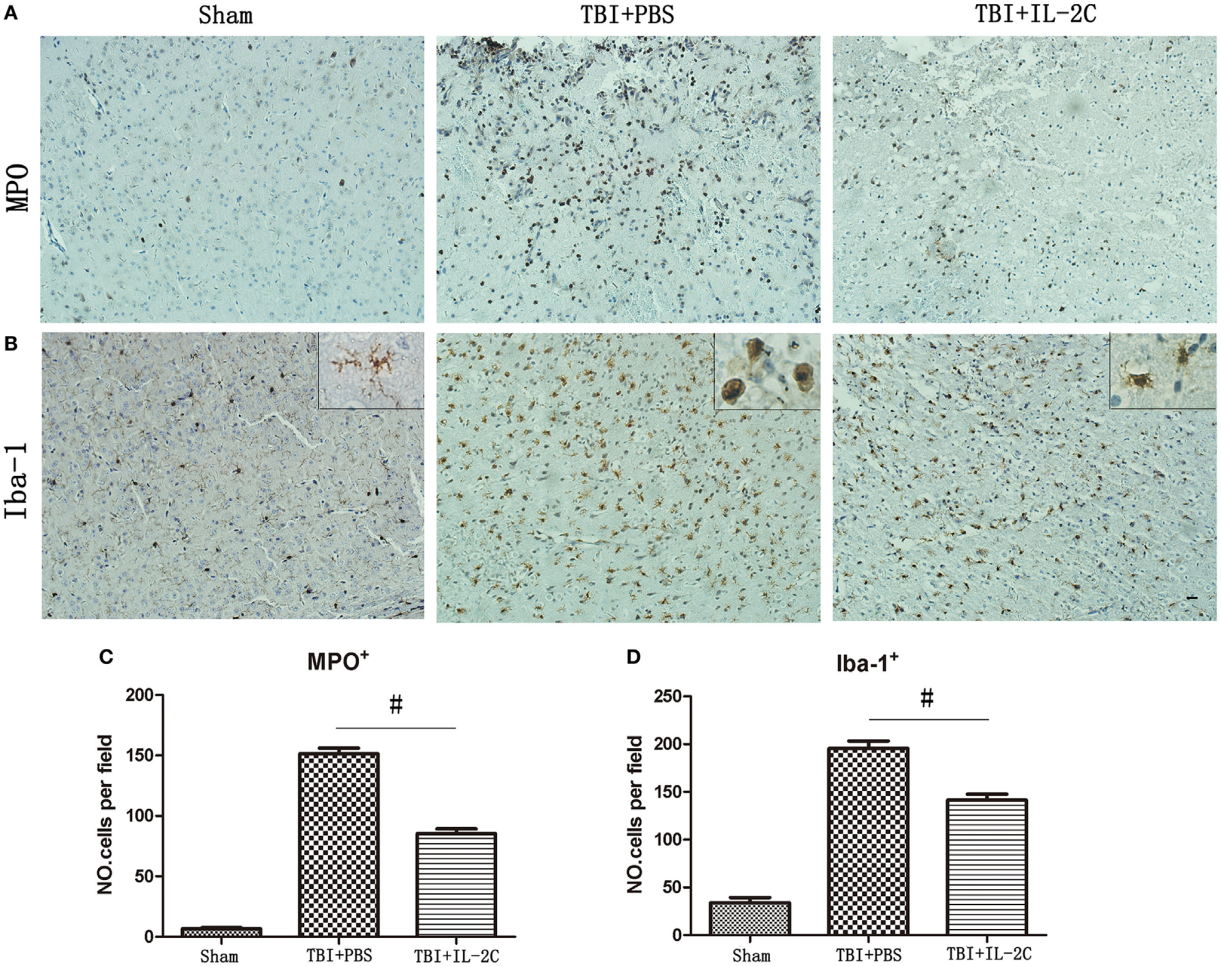
**(A,C)** IL-2C administration significantly reduced infiltration of neutrophils in the brain at day 3 after traumatic brain injury (TBI) (*n* = 5 mice/group). **(B,D)** Treatment with IL-2C resulted in significantly fewer activated microglia in the cortical contusion margin on day 3 post-injury (*n* = 5 mice/group). And morphological observation showed that TBI provoked a drastic change in the morphology of microglia from the elongated and ramified shape to a round and enlarged appearance. And treatment with IL-2C significantly reduced the soma size and ramification index. Data are presented as the mean ± SD; *p < 0.05 and ^#^*p* < 0.01.

Brain injury induces infiltration of neutrophils and activation of microglia. To determine whether treatment with IL-2C affected these inflammatory cells, we used immunohistochemistry to count the numbers of microglia and neutrophils. We observed that administering IL-2C resulted in fewer MPO^+^ cells than were observed in the PBS-treated mice (*n* = 85.40 ± 8.44 versus 151.40 ± 10.64/field; *p* < 0.01) at 3 days post-injury (Figures 9A,C). There were also fewer Iba-1^+^ cells in the peri-contusion area on day 3 post-injury in the IL-2C-treated group (*n* = 195.6 ± 7.64 versus 141.4 ± 6.27/field, *p* < 0.01) (Figures 9B,D).

Then, we also observed the morphometric changes of microglia in different condition. And we observed that TBI provoked a drastic change in the morphology of microglia from the elongated and ramified shape to a round and enlarged appearance, suggestive of massive activation. And IL-2C treatment significantly reduced the soma size and ramification index (Figure 9B).

### Effects of IL-2C Administration on the Pro-inflammatory Responses

**Figure 10:**
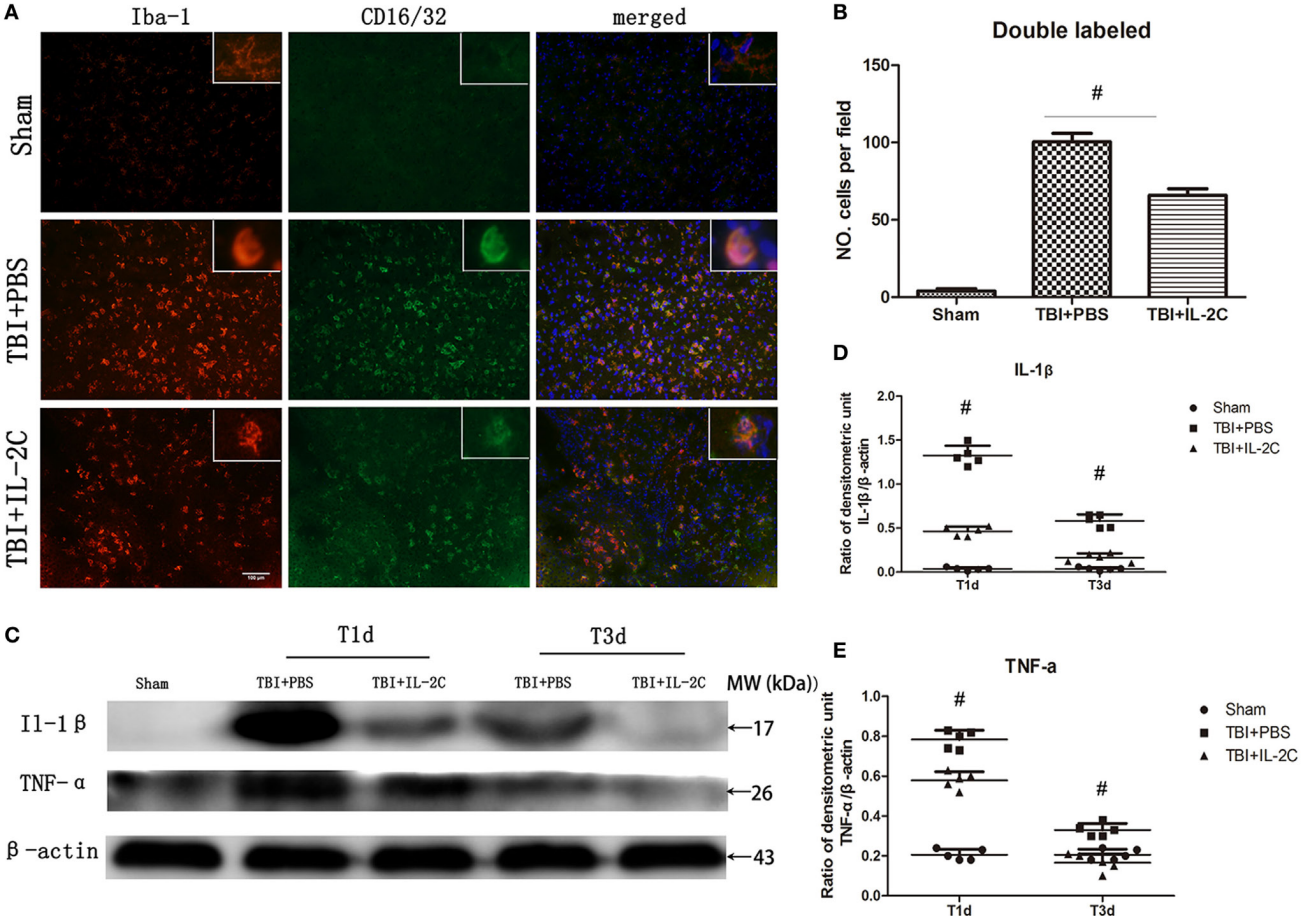
Post-injury IL-2C administration suppressed the expression of M1-associated markers around the lesion area. **(A)** Co-localization of CD16/32 with the microglia marker Iba1 in the contusion area on day 3 post-injury showed that treatment with IL-2C markedly reduced the amount of M1 microglia. **(B)** Cell count analyses indicated that IL-2C significantly reduced the number of CD16/32 and Iba-1 double-positive cells. **(C–E)** The traumatic brain injury (TBI)-induced expression of IL-1β and TNF-α in the ipsilateral cortices on days 1 and 3 post-injury was inhibited by treatment with IL-2C. Data are presented as the mean ± SD; *p < 0.05 and ^#^*p* < 0.01 (*n* = 5 mice/group).

The M1 or M2 polarization of microglia is commonly characterized by the expression of surface makers associated with the M1 or M2 phenotype. It was previously reported that brain injury significantly increased the expression of M1 phenotype, which generally release pro-inflammatory cytokines. We co-labeled CD16/32 with Iba-1 to determine the frequency of M1 cells and found that TBI led to a robust increase in the expression of double-labeled cells (100.4 ± 5.59 versus 4.0 ± 1.58/field, *p* < 0.01), while treatment with IL-2C significantly reduced the expression of these cells in the peri-contusion area (65.8 ± 4.21 versus 100.4 ± 5.59/field, *p* < 0.01) (Figures 10A,B). Moreover, using western blot analysis, we found that the expression of IL-1β and TNF-α, which are pro-inflammatory cytokines mainly released by M1 microglia, were significantly elevated in the ipsilateral cortices. Treatment with IL-2C significantly reduced the levels of IL-1β (day 1: 1.32 ± 0.11 versus 0.46 ± 0.05%, *p* < 0.01; day 3: 0.58 ± 0.07 versus 0.16 ± 0.05%, *p* < 0.01) and TNF-α (day 1: 0.78 ± 0.05 versus 0.58 ± 0.04%, *p* < 0.01; day 3: 0.33 ± 0.03 versus 0.17 ± 0.04%, *p* < 0.01) (Figures 10C–E).

### Effects of IL-2C Administration on the Anti-inflammatory Responses

**Figure 11:**
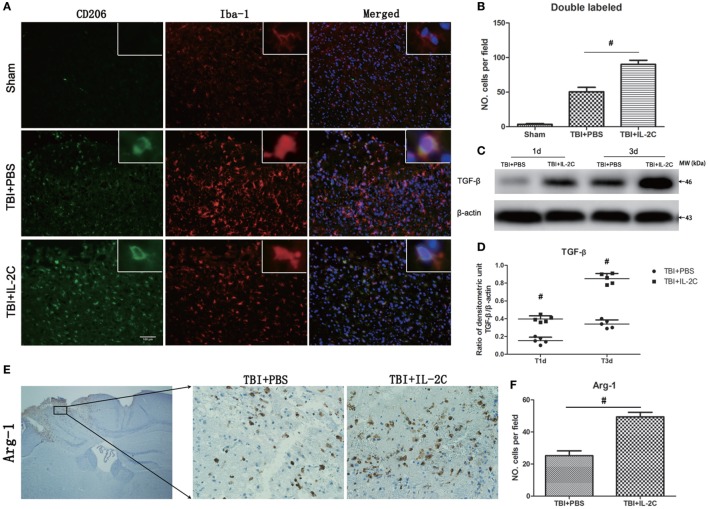
Treatment with IL-2C increased the expression of M2-associated markers in the contusion margin. **(A)** Co-localization of CD206 with the microglia marker Iba1 showed that IL-2C increased the number of M2 microglia on day 3 post-injury in the contusion area. **(B)** Cell count analyses indicated that IL-2C significantly increased the amount of CD206 and Iba-1-double-positive cells. **(C,D)** The traumatic brain injury TBI-induced expression of TGF-β in the ipsilateral cortices on days 1 and 3 post-injury was increased by treatment with IL-2C. **(E,F)** Immunohistochemistry showed that treatment with IL-2C significantly increased the number of arginase-1^+^ cells around the lesion area. Data are presented as the mean ± SD; *p < 0.05 and ^#^*p* < 0.01 (*n* = 5 mice/group).

We next assessed the effects of IL-2C on the frequency of M2 phenotype. We observed that IL-2C significantly elevated the number of Iba-1/CD206 (50.6 ± 6.39 versus 90.2 ± 5.89, *p* < 0.01) and Arg-1 (25.2 ± 3.03 versus 49.4 ± 2.88, *p* < 0.01) in the peri-contusion area compared to the PBS-treated group (Figures 11A,B,E,F). TGF-β is an important anti-inflammatory cytokine described to decrease the cytotoxic properties of activated microglia. And treatment with IL-2C significantly increased the expression of TGF-β (day 1: 0.15 ± 0.04 versus 0.29 ± 0.03%, *p* < 0.01; day 3: 0.22 ± 0.03 versus 0.85 ± 0.06%, *p* < 0.01) (Figures 11C,D).

## Discussion

In this study, we used CCI device to prepare TBI models. Unlike the model prepared by the fluid percussion injury device, which deliver a fluid pulse to the intact dura to create a diffuse injury (25, 26), the CCI model creates focal damage with a localized injury. The main advantage of this device is that the deformation parameters (i.e., velocity, depth, and time of duration) could be precisely controlled, making TBI models highly reproducible (27). Besides, CCI could mimic the whole spectrum of focal injury, and translate well to human TBI. In present study, we mainly observed the effects of IL-2C on inflammation and blood–brain barrier disruption in the cortex ipsilateral to injury.

Tregs are special subtype of T cells that play a critical role in suppressing inflammatory responses and maintaining immune homeostasis. Yu et al. showed that depleting Tregs using anti-CD25 antibodies aggravates the inflammatory response, whereas the adoptive transfer of Tregs reduces the damage caused by inflammation. Despite this evidence, the use of adoptive transfer is limited by the lack of a method to obtain Tregs in sufficient numbers and purity and the consequent need to expand these cells *in vitro* to achieve sufficient numbers for transfer. Thus, we describe an alternative method for performing adoptive transfer that involves using low doses of IL-2 complexed with anti-IL-2 to induce the selective *in vivo* expansion of Tregs.

TLR4 is an important member of the TLR family, and high expression of TLR4 has been observed in the tissue around brain trauma. Ahmad et al. showed that TLR4 knockout reduces the development of neuroinflammation, tissues injury events associated with brain trauma, suggesting that therapies targeting TLR4 have great potential in improving the prognosis of TBI (28). Then, TLR4 interacts with its downstream receptor, which subsequently phosphorylates and decomposes IκB. Then, NF-κB separates from IκB and translocates into the nucleus to trigger the transcription of multiple inflammatory genes, upregulating the expression of a series of inflammatory cytokines, such as IL-1β, TNF-α, and iNOS. NF-κB is the master regulator of a series of inflammatory cytokines and is significantly elevated in the tissue surrounding brain trauma, and specific NF-κB inhibitors significantly reduced the expression of inflammatory cytokines after TBI and relieved secondary brain injury (29). Thus, in this study, we selected both TLR4 and NF-κB indicators to assess the effects of different IL-2C doses. We observed that IL-2C dose-dependently increased the number of Tregs. And conventional dose of IL-2C increased the proportion of Tregs within the CD4^+^ population by approximately fourfold in the spleen, whereas dramatically upregulated the TLR4 and NF-κB p65. And half dose could suppress the inflammatory responses while increasing the proportion of Tregs by approximately twofold. One reason for this may be that excess Tregs deprive the body of the beneficial effects of a moderate immune-inflammatory response, which is necessary for physiological function, contributing to the repair of injury tissue (30, 31).

The treatment with IL-2C could increase the number of Tregs in the ipsilateral cortices using flow cytometry analysis. The increased Tregs were associated with reduced cerebral edema, lesion volume, and improved neurological functions recovery. These beneficial effects may be mediated by the reduction of BBB disruption and pro-inflammatory responses, and the increase of anti-inflammatory responses.

The BBB is crucial for maintaining homeostasis in the CNS (32, 33). Key components of BBB are the cerebral blood vessels, which are formed by ECs. ECs possess continuous intercellular TJs that limit both the paracellular and transcellular movement of molecules through the EC layer (34). TBI disrupts the BBB and causes damage by disrupting tight junction complexes, widening intercellular spaces, flattening and compressing the vasculature and reducing the vascular lumen, all of which contribute to cellular swelling (35). The disrupted BBB then allows circulating cells and many blood-borne substances into the brain. Excessive accumulation of these leukocytes results in the release of large amounts of cytokines (i.e., cytotoxic enzymes, inflammatory mediators, and reactive oxygen species), which further damages the microvascular endothelium and TJs. In this study, treatment with IL-2C increased the expression of TJs (ZO-1, occluding, and claudin-5), suggesting that the increase in Tregs may be able to maintain the integrity of BBB. Then, the relatively intact barrier reduced the infiltration of leukocytes into the central nervous system, just as we observed.

Microglia is an important component of innate immunity and plays essential roles in the inflammatory response (36). The microglial activation is associated with enhanced phagocytosis and the upregulation of various immunomodulatory factors, as well as the secretion of inflammatory and cytotoxic molecules leading to secondary neuronal damage (37, 38). Attenuating the over-activation of microglia was shown to protect the brain from inflammatory lesion (39). In addition, microglial cells are of high plasticity, would change their morphology after TBI. Reports showed that quiescent microglia exhibit a ramified cell morphology with relatively small soma and numerous thin branches, whereas activated microglia become progressively less ramified and quickly develop an enlarged cell body with several short, thickened branches, resulting in a rounded amoeboid-like appearance (40, 41). In this study, we showed that treatment with IL-2C could significantly reduce the microglial over-activation and the soma size and ramification index of microglia.

As already demonstrated, microglia have two different phenotypes—classically activated (M1) and alternatively activated (M2) microglia (42). An accumulating amount of evidence suggests that M1 microglia are a pro-inflammatory subtype that is associated with tissue destruction, whereas M2 microglia are an anti-inflammatory subtype that facilitates repair and regeneration (21, 43, 44). TBI activates both the classic M1 and alternative M2 phenotype. However, M2 activity decreases within a few days after injury, whereas M1 activity remains elevated much longer and intensifies damage following TBI over time (45, 46). Furthermore, microglia may shift from one phenotype to another in a specific microenvironment (47). All indicated that M2-polarized microglia may resolve the excessive inflammation and promote tissue repair (36, 48). In this study, we assessed the cerebral polarization of microglia by analyzing the M1 or M2 phenotypes in the contusion margin. For immunofluorescence, we co-labeled CD16/32 or CD206 with Iba-1 to define the M1 or M2 phenotype of microglia, because only one maker could not determine the phenotype of microglia. We observed that TBI activated plenty of microglia, resulting in a significantly increase in the expression of M1 associated markers (i.e., CD16/32, IL-1β, TNF-α). And treatment with IL-2C reduced the expression of M1-associated markers while increasing the expression of M2-associated markers (i.e., CD206, arginase 1, TGF-β). Our data clearly indicate that treatment with IL-2C promoted the M2 polarization of microglia/macrophages in the ipsilateral cortices.

In summary, treatment with IL-2C significantly increased the number of Tregs in a dose-dependent manner. Unlike what has been observed in other diseases, conventional dose was harmful to wound healing in this model, while a half-dose could reduce the brain edema, tissue loss, and promote the neurological function recovery. Moreover, IL-2C administration also could alleviate the degradation of BBB, reduce the expression of M1-associated makers, and increase the expression of M2-associated makers. Besides, a limitation exists in this study, and further investigations will be required to assess the potential effects of IL-2C in sham animals.

## Ethics Statement

This study was carried out in accordance with the recommendations of the Animal Care and Ethics Committee of Tianjin Medical University General hospital, China. The protocol was approved by the Animal Care and Ethics Committee of Tianjin Medical University General hospital, China.

## Author Contributions

WG, FL, and ZZ are the co-first authors. WG: designed the study, performed the TBI model and flow cytometry, and wrote the manuscript. FL and ZZ: prepared the drug solutions and performed histological examination and western blot. XX and YW: performed the western blot and the animal studies. SZ and DY: detect the water content. DS and JX: evaluate the beam walk and forelimb foot faults. RJ and JZ: contributed to the design and analysis of the study and wrote the manuscript. All authors approved the final version of the manuscript.

## Conflict of Interest Statement

The authors declare that the research was conducted in the absence of any commercial or financial relationships that could be construed as a potential conflict of interest.
